# Supplementary material for: The interactome of intact mitochondria by cross-linking mass spectrometry provides evidence for coexisting respiratory supercomplexes
Source: Mol Cell Proteomics. 2017 Dec 8;17(2):216–32. doi: 10.1074/mcp.RA117.000470 (PMC5795388; doi:10.1074/mcp.RA117.000470)
Supplement: Supplemental Data [file supp_RA117.000470_133922_0_supp_23978_fzffwf.zip › spectra_annotation/mito_spectra_annotation/82-2-2-1-6-1.pdf]

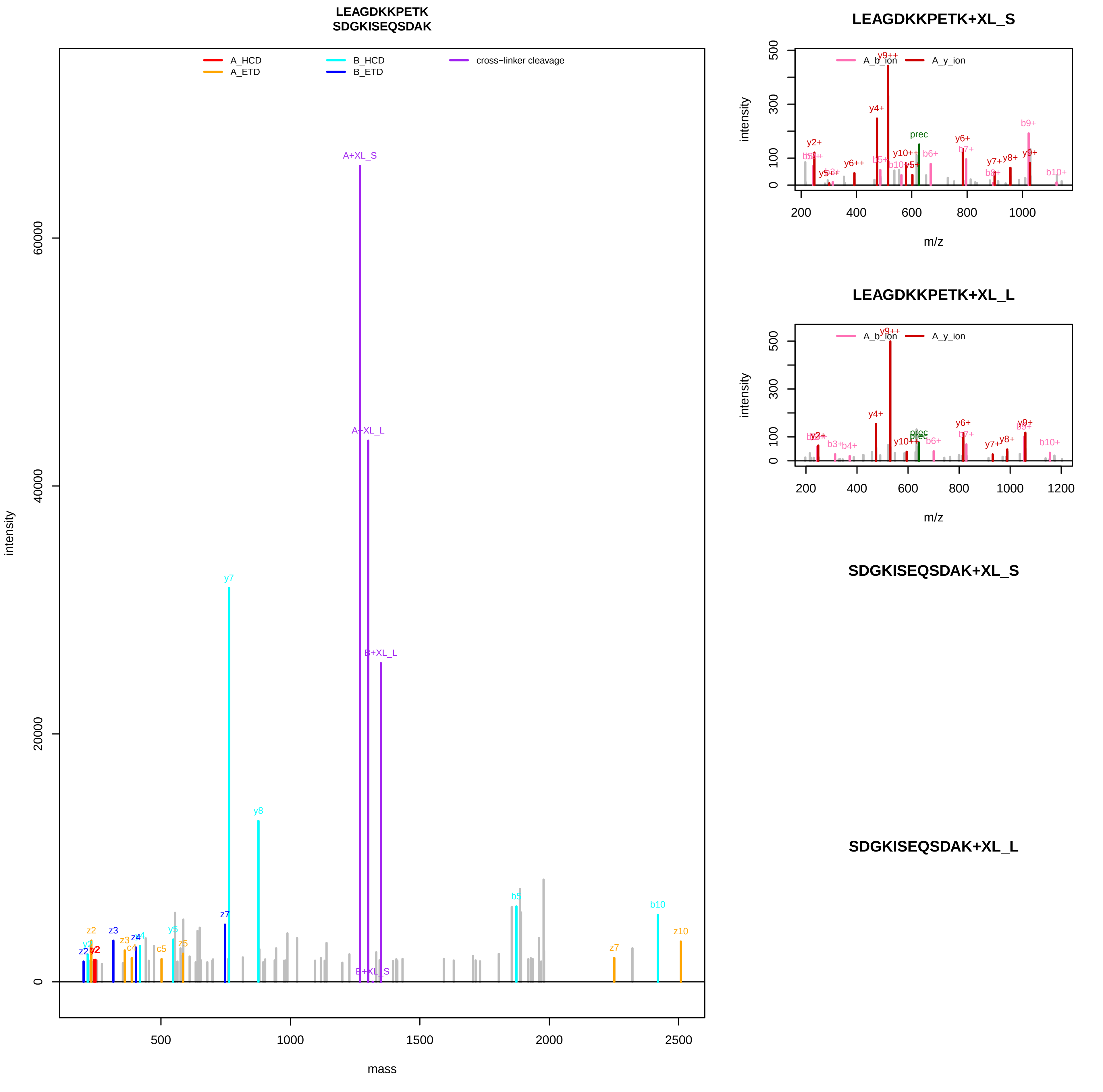
LEAGDKKPETK
SDGKISEQSDAK
A_HCD
A_ETD
B_HCD
B_ETD
cross−linker cleavage
A+XL_S
A+XL_L
B+XL_L
B+XL_S
y7
y8
z7
b5
b10
z10
z7
intensity
mass
0
20000
40000
60000
500
1000
1500
2000
2500
LEAGDKKPETK+XL_S
LEAGDKKPETK+XL_L
SDGKISEQSDAK+XL_S
SDGKISEQSDAK+XL_L
A_b_ion
A_y_ion
y9++
y4+
prec
y6+
b7+
b9+
y9+
y8+
y7+
b10+
b6+
y2+
y6++
m/z
200
400
600
800
1000
1200
0
100
300
500
